# Characterization of Neuraminidases from the Highly Pathogenic Avian H5N1 and 2009 Pandemic H1N1 Influenza A Viruses

**DOI:** 10.1371/journal.pone.0015825

**Published:** 2010-12-29

**Authors:** Jia Wu, Fengwei Zhang, Maorong Wang, Chunqiong Xu, Jingdong Song, Jianfang Zhou, Xiaojing Lin, Yonghui Zhang, Xiaobing Wu, Wenjie Tan, Jian Lu, Honglan Zhao, Jimin Gao, Ping Zhao, Jianxin Lu, Yue Wang

**Affiliations:** 1 Key Laboratory for Laboratory Medicine, Ministry of Education, Zhejiang Provincial Key Laboratory of Medical Genetics, School of Laboratory Medicine and Life Science, Wenzhou Medical College, Wenzhou, People's Republic of China; 2 Liver Disease Center of P.L.A., the 81st Hospital of PLA, Nanjing, People's Republic of China; 3 School of Clinical Sciences, NingXia Medical University, Yinchuang, People's Republic of China; 4 State Key Laboratory for Molecular Virology and Genetic Engineering, National Institute for Viral Disease Control and Prevention, China Center for Disease Control and Prevention, Beijing, People's Republic of China; 5 Department of Microbiology, Shanghai Key Laboratory of Medical Biodefense, The Second Military Medical University, Shanghai, People's Republic of China; University of Georgia, United States of America

## Abstract

To study the precise role of the neuraminidase (NA), and its stalk region in particular, in the assembly, release, and entry of influenza virus, we deleted the 20-aa stalk segment from 2009 pandemic H1N1 NA (09N1) and inserted this segment, now designated 09s60, into the stalk region of a highly pathogenic avian influenza (HPAI) virus H5N1 NA (AH N1). The biological characterization of these wild-type and mutant NAs was analyzed by pseudotyped particles (pseudoparticles) system. Compared with the wild-type AH N1, the wild-type 09N1 exhibited higher NA activity and released more pseudoparticles. Deletion/insertion of the 09s60 segment did not alter this relationship. The infectivity of pseudoparticles harboring NA in combination with the hemagglutinin from HPAI H5N1 (AH H5) was decreased by insertion of 09s60 into AH N1 and was increased by deletion of 09s60 from 09N1. When isolated from the wild-type 2009H1N1 virus, 09N1 existed in the forms (in order of abundance) dimer>>tetramer>monomer, but when isolated from pseudoparticles, 09N1 existed in the forms dimer>monomer>>>tetramer. After deletion of 09s60, 09N1 existed in the forms monomer>>>dimer. AH N1 from pseudoparticles existed in the forms monomer>>dimer, but after insertion of 09s60, it existed in the forms dimer>>monomer. Deletion/insertion of 09s60 did not alter the NA glycosylation pattern of 09N1 or AH N1. The 09N1 was more sensitive than the AH N1 to the NA inhibitor oseltamivir, suggesting that the infectivity-enhancing effect of oseltamivir correlates with robust NA activity.

## Introduction

Influenza A viruses cause seasonal epidemics and occasional pandemics [1], [2], [3]. The outbreak of a novel H1N1 influenza strain became a major global issue in April 2009 and, to date, this virus, here designated 2009H1N1, has been detected in 214 countries and has caused 17,919 deaths [4]. In addition, a highly pathogenic avian influenza (HPAI) H5N1 virus has been circulating in Europe and Asia for more than a decade and has spread to more than 60 countries; thus far, it has infected 486 humans and killed 287 of them [5]. Although reports of human-to-human HPAI H5N1 transmission are rare, its high lethality has raised considerable concern worldwide.

Influenza viruses contain eight negative-sense single-stranded RNA segments that together encode 11 proteins [2]. Two of these proteins, hemagglutinin (HA) and neuraminidase (NA), are large glycoproteins found on viral envelope [1], [2], [6]. HA mediates binding of the virus to host cell receptors and promotes entry of the viral genome into the target cell through membrane fusion, whereas NA cleaves terminal sialic acids from oligosaccharide side-chain receptors that bind the mature progeny virus particles, thereby releasing them from infected cells, and regulates virus entry [2]. HA and NA are also human antigens; the host immune responses to these proteins are used to classify influenza A viruses into 16 HA subtypes and nine NA subtypes (hence the “H#N#” designation for influenza A serotypes) [1], [2]. HA is a trimeric, rod-shaped molecule that is anchored in the viral membrane by its carboxyl (C)-terminus; cleavage of the HA precursor molecule (HA0) into two subunits (HA1 and HA2) is required for full activity [1], [2], [7], [8]. NA is a mushroom-shaped tetramer of monomers that contain four structural domains: a cytoplasmic domain, a transmembrane domain, a stalk, and a globular head [2], [9], [10], [11]. The evolution of influenza HA and NA proteins has been monitored closely in recent years and has revealed significant variation in the NA stalk region [12]–[14]. Based on their sequences, NA stalk regions have been divided into six types [15]. Compared to the A/Gs/Gd/1/96/H5N1-like stalk region, the A/WSN/33/H1N1-like stalk region has a 16-amino acid (aa) deletion of residues 57–72, A/Puerto Rico/8/34/H1N1-like has 15-aa deletion of residues 63–77, A/Hong Kong/156/97/H5N1-like has a 19-aa deletion of residues 54–72, A/chicken/Italy/1067/99/H7N1-like has 22-aa deletion of residues 54–75, and A/chicken/Hubei/327/2004/H5N1-like has a 20-aa deletion of residues 49–68 [15]. The extent of these deletions appears to have increased gradually; however, the biological impacts of variations in the NA stalk are not yet clear.

Studies of the A/WSN/33/H1N1 strain have shown that its biology has not been altered significantly by accumulating deletions and insertions [11]. However, recent studies have indicated that the NA stalk plays a critical role in viral replication, virulence, pathogenesis, and species adaptation [9], [15], [16]. Our previous studies showed that the NA activity of A/Ohio/07/2009/H1N1 was much higher than that of HPAI H5N1 (A/Anhui/1/2005) and that this increased activity is a key reason that the NA inhibitor oseltamivir enhances the infectivity of 2009H1N1 [17].

In our previous study, a primary sequence alignment revealed a 20-aa (60-bp) deletion in the stalk region of HPAI H5N1 (A/Anhui/1/2005) NA relative to that of 2009H1N1 NA [17]. Here, we examined the precise role of NA, and particularly its stalk region, in influenza A virus assembly, release, and entry, by deleting the 20-aa segment (residues 49–68) from the stalk region of 2009H1N1 NA, and inserted this segment, designated 09s60, into the stalk region of H5N1 NA. We then made HA/NA pseudoparticles containing all possible combinations of each HA with each wild-type and mutant NA. The pseudoparticles were evaluated by transmission electron microscopy, virion quantification; hemagglutination, NA, and infectivity assays; and Western blot analysis of HA and NA expression in pseudoparticles producer cells and incorporation into pseudoparticles. The effect of oseltamivir on these pseudoparticles was also assessed.

## Materials and Methods

### Cell culture

For pseudotyping, A549 targeting cells and 293T human embryo kidney pesudoparticle producer cells were obtained from American Type Culture Collection (Manassas, VA) and grown in Dulbecco's modified essential medium (DMEM; Invitrogen, Carlsbad, CA) supplemented with or without 10% fetal bovine serum.

### Construction of NA and HA expression plasmids

**Figure 1:**
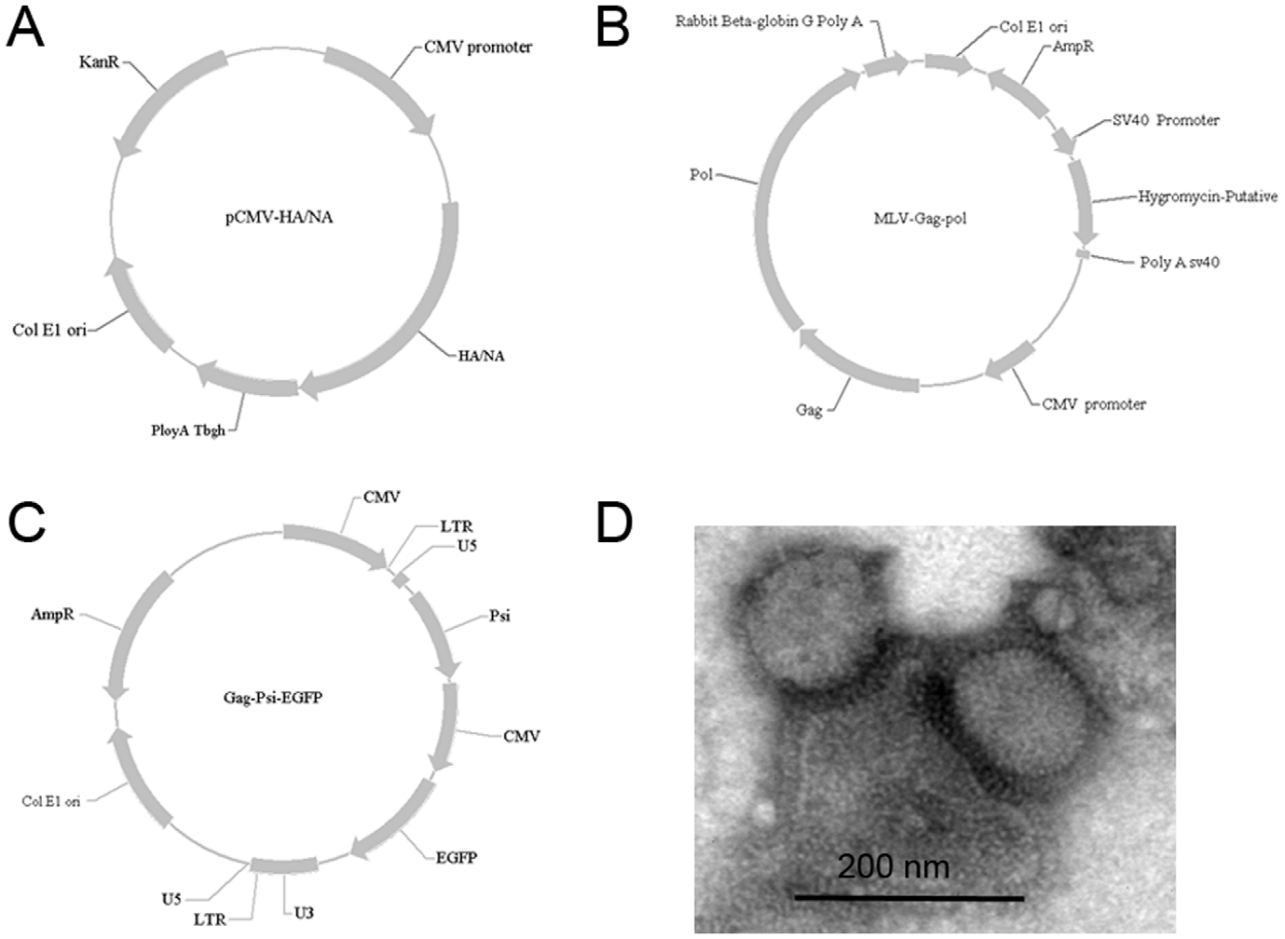
Diagrams of HA/NA expression plasmids, GagPol-encoding plasmid, and CMV–GFP reporter plasmid, and transmission electron microscope observation. A. HA/NA expression plasmids, HA/NA encoding sequences were inserted between the CMV promoter and poly A Tbgh (bovine growth hormone) tail. B. Murine leukemia virus GagPol-encoding plasmid, GagPol-encoding sequence was inserted between the CMV promoter and the Rabbit beta-globin G ploy A tail. C. CMV–GFP reporter plasmid, the EGFP sequence was inserted into the U3 and U5 sequence of murine leukemia virus. D. Transmission electron microscopy image of pseudoparticles.

The cDNA fragments encoding full-length NA (A/Ohio/07/2009, the most early released NA sequence of 2009 H1N1 which without aa difference with that of A/California/05/2009/H1N1 isolate) and HA (A/California/05/2009) proteins from 2009 pandemic H1N1 and HPAI H5N1 (A/Anhui/1/2005) were cloned into the expression plasmids (Figure 1A) as reported previously [17]–[21]. The H5N1-derived HA and NA were designated AH H5 and AH N1, respectively, and the 2009H1N1-derived HA and NA were designated 09H1 and 09N1, respectively. One of the notable differences between the H5N1 and 2009H1N1 NA proteins was that the 09N1 contained a 20-aa (60-bp) “insertion” in its stalk region relative to the AH N1 stalk region. This 20-aa segment of 09N1, designated s60 or 09s60, was deleted from 09N1 to generate 09N1-s60 and inserted into AH N1 to generate AH N1+09s60. Due to lacking of antibody against AN NA, a 6×His-coding sequence [(ATG)_6_] was added to the C-terminal ends of the AH N1 and AH N1+09s60 coding sequences to facilitate Western blot analysis.

### Pseudoparticle production and quantification

As we reported previously [17]–[21], pseudoparticles were produced by transfecting 293T cells with four plasmids: an HA expression plasmid, an NA expression plasmid, a GagPol-encoding plasmid, and a cytomegalovirus (CMV)–green fluorescent protein (GFP) reporter plasmid (for details, please see Figure 1A, B, and C). Eight types of pseudoparticles were made. Those containing HA and NA from the same virus (*e.g*., AH H5::AH N1 and 09H1::09N1) were considered “native,” and those containing HA and NA of different viruses (*e.g.*, AH H5::09N1 and 09H1::AH N1) were considered “mismatched”. A pseudoparticle containing a mutant NA could be either a “native mutant” (*e.g.*, AH H5::AH N1+09s60) or a “mismatched mutant” (*e.g.*, AH H5::09N1-s60), depending on whether the AH and NA came from the same (native) or different (mismatched) viruses.

At 72 h post-transfection, pseudoparticles were harvested from the transfected cell culture media by filtration through a 0.45-µm Durapore polyvinylidene fluoride (PVDF) membrane filter (Millipore Ireland, Cork, Ireland). For quantification, purified pseudoparticles were treated with 0.24 U/ml DNase and RNase at 37°C for 1 h to eliminate any contaminating DNA and RNA and then frozen at –70°C to inactivate the DNase and RNase. The pseudoparticles were then treated with proteinase K (Qiagen, Valencia, CA) at 40°C for 30 min to digest the envelope proteins and release the CMV–GFP RNA. The proteinase K was inactivated at 100°C for 2 min.

The amount of CMV–GFP RNA in each pseudoparticle was quantified by real-time quantitative reverse-transcription (qRT)–PCR using the forward primer 5′-CCCGTGAGTCAAACCGCTAT-3′, the reverse primer 5′-GTGATGCGGTTTTGGCAGTA-3′, and the probe 5′-FAM-CCACGCCCATTGATG-NFQ-3′, where FAM is the fluorescent dye 6-carboxy-fluorescein and NFQ is a non-fluorescent quencher. The assay was carried out on an ABI 7500 Fast Real-Time PCR System (Applied Biosystems, Foster City, CA) with a PrimeScript One Step RT–PCR Kit (Takara, Japan). Pseudoparticles were normalized for RNA copy number before infectivity, hemagglutination, and NA activity assays, and Western blot analysis of the HA and NA expression and incorporation.

### Transmission electron microscopy

The supernatant of the medium containing pseudoparticles were concentrated at 40,000 rpm for 25 min in a Hitachi centrifuge (Hitachi, Japan). Subsequently, both the original and concentrated supernatants were negative stained, and the pseudoparticles were observed using transmission electron microscopy (TECNAI 12, FEI, Blackwood, NJ) with an acceleration voltage of 80 kV.

### Infectivity assay

Pseudoparticles were assayed for infectivity as reported previously [17]–[21]. Briefly, pseudoparticles (normalized for RNA copy number) were diluted 1:1 in 100 µL of DMEM. To infect cells, the cell culture medium was removed and replaced with the diluted pseudoparticle suspension. The plate was allowed to stand for 4 h, and the pseudoparticle suspension was removed and replaced with DMEM supplemented with 10% fetal bovine serum. At 72 h post-infection, the infected cells were rinsed twice with phosphate-buffered saline (PBS). The number of GFP reporter-positive cells was determined by fluorescence-activated cell sorting (BD FACSAria; BD Biosciences, Franklin Lakes, NJ).

We previously reported that oseltamivir inhibits viral NA activity and pseudoparticle release while boosting viral infectivity *in vitro* [17], [20]. Here, to examine the relationship between NA activity and the infectivity-enhancing effect of oseltamivir, we performed parallel infectivity assays on the pseudoparticle suspensions in the presence of 0.25 µM oseltamivir.

### Hemagglutination assay

The pseudoparticles were assayed for hemagglutination activity. The normalized pseudoparticle samples were serially diluted 1∶2 in PBS in microplate wells (50 µL/well). Next, 50-µL aliquots of turkey red blood cells (1% suspension) were added to each well. Hemagglutination was scored 30 min later.

### NA activity assay

The NA activity of the 09N1, 09N1-s60, AH N1, and AH N1+09s60 proteins was assayed using an NA-Star Influenza Neuraminidase Inhibitor Resistance Detection Kit (Applied Biosystems). All reagents were prepared according to the manufacturer's instructions. For each sample, normalized pseudoparticles were diluted 1∶1 in NA-Star Assay buffer, incubated with 10 µL of NA-Star chemiluminescent substrate for 30 min at room temperature, and analyzed using a luminometer (2103 Envision Multilabel Reader; Perkin-Elmer, Waltham, MA).

### Western blot analysis of HA and NA expression and incorporation into pseudoparticles

The expression of HA and NA in 293T producer cells was analyzed after pseudoparticle harvesting. Cells in 6-well plates were washed twice with PBS to remove residual culture medium and then lysed in a 500 µL/well of reporter lysis buffer (Promega, Madison, WI). After centrifugation to remove cell debris, 20 µL of each cell lysate sample was mixed with 4× LDS Sample Buffer (Invitrogen), heated to 100°C for 5 min, and analyzed by Western blotting as described below.

To evaluate HA and NA incorporation into pseudoparticles and to assess the possible influence of NA mutations as well as partner mismatches on HA incorporation, the pseudoparticles were concentrated and purified using a standard protocol. Then, 10^9^ copies of each pseudoparticle were mixed with 4× LDS Sample Buffer (Invitrogen), heated to 100°C for 5 min, and analyzed by Western blotting as described below.

For Western blot analysis, the prepared cell lysate and pseudoparticle samples (in non-reduced condition) were subjected to electrophoresis on 12% NuPage gels (Invitrogen). After electrophoresis, the separated proteins were transferred to PVDF membranes (Pall Corp., Port Washington, NY) using a semi-dry transfer method. For HA and NA detection, the membranes were blocked with skim milk overnight at 4°C. They were then incubated with a primary antibody against 09H1 (Sino Biological Inc, Beijing, China), AH H5 (Dr. Mifang Liang), 09N1 (AbMax Biotechnology Co., Ltd, Beijing, China), or 6×His-Tag (mouse monoclonal antibody from CWBIO, Beijing, China) for 1.5 h at room temperature. After three washes in PBS, the membranes were incubated with a biotinylated secondary antibody (Vector Laboratories, Burlingame, CA) for 20 min at room temperature and washed three times in PBS. NA and HA were then visualized using a DAB Substrate Kit (Vector Laboratories).

### Data analysis

Differences were evaluated using a two-tailed Fisher's exact test (SPSS, release 12.1; SPSS Inc., Chicago, IL). Differences were considered statistically significant at *P*<0.05.

## Results

### Amino acid sequence alignments

**Figure 2:**
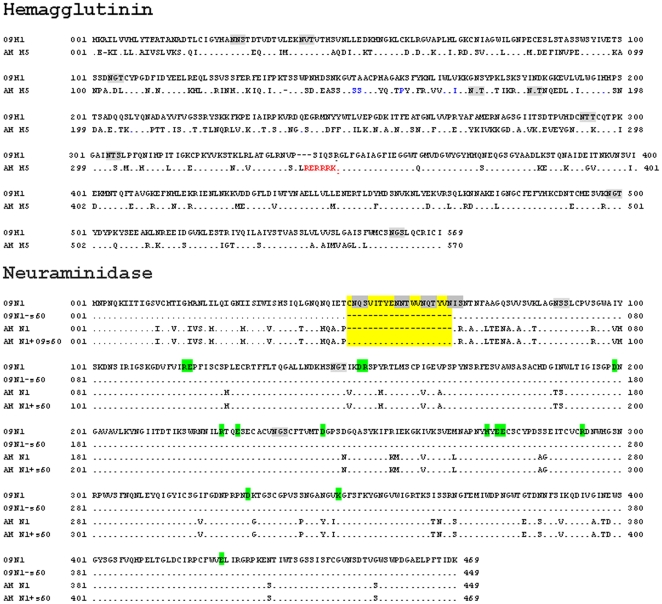
Alignment of influenza virus A hemagglutinin (HA; upper) and neuraminidase (NA; lower) sequences to those of the 2009 pandemic H1N1 strain (2009H1N1). Dots indicate residues that are identical to the corresponding residues in 2009H1N1, and dashes indicate deleted residues. Signal peptides are shown in bold type. Gray boxes indicate potential glycosylation sites, as predicted from the amino acid sequence. For HA, red residues represent the precursor cleavage site that links the functional HA1 and HA2 domains, and blue residues represent the binding site for the sialic acid receptor. For NA, conserved active site residues are highlighted in green, and the 20-aa insertion/deletion segment of interest in the stalk region is highlighted in yellow.

We compared the amino acid sequences of the HA proteins from the 2009 pandemic influenza strain A/California/05/2009/H1N1 (GenBank #FJ966952) and influenza A/Anhui/1/2005/H5N1 (#DQ371928). As shown in Figure 2 (upper panel), these two HA sequences, designated 09H1 and AH H5, respectively, differ at 207 of 569 residues (36.4%), exhibiting considerable variation in their glycosylation, receptor binding, and cleavage sites.

We also compared the sequences of the NA proteins from influenza A/Ohio/07/2009/H1N1 (#FJ969534) and influenza A/Anhui/1/2005/H5N1. As shown in Figure 2 (lower panel), these two NA sequences, designated 09N1 and AH N1, respectively, differ at 55 of 469 residues (11.7%). In particular, the 09N1 stalk region contains a 20-aa segment (designated s60 or 09s60; highlighted in yellow) that is not present in AH N1. This gap in the AH N1 stalk region sequence results in the loss of four potential glycosylation sites (gray boxes) and a cysteine residue from AH N1. Of the 29 aas in the transmembrane domains, there are 8 aas difference (27.5%) which is much higher than the that of other regions, As previously noted, the 15 charged active site residues are conserved [18], [22], [23].

To examine the precise function of the NA stalk region in viral assembly, entry, and release, we deleted the 20-aa stalk segment from 09N1 to create 09N1-s60, and we inserted this s60 segment into AH N1 to create AH N1+09s60.

### Transmission electron microscope observation

To verify the pesudoparticles, we observed it using transmission electron microscopy. The pesudoparticles were visible as a particle surrounded with HA and NA spikes, which matches the typical viral morphology of influenza virus (Figure 1D). Meanwhile, no typical viral morphology of influenza virus was found neither in the supernatant from naïve 293T cell nor in the supernatant from 297T cell transfected with a GagPol-encoding plasmid and a CMV–GFP reporter plasmid but no HA and NA expression plasmids. Together with the following serial functional assessment on both HA and NA of pseudoparticles, what we observed is the functional HA/NA bearing pseudotyped influenza viruses particle.

### The pseudoparticle-releasing ability of 09N1 is stronger than that of AH N1, independent of the stalk region insertion

By removing terminal sialic acids from the oligosaccharide side chains to which viral HA binds, NA plays a central role in the release of the virus from infected cells [2]. To compare the virus-releasing ability of 09N1 and AH N1, and to examine the role of the stalk region in this function, we generated eight pseudoparticles by combining each of the two HAs (AH H5 and 09H1) with each of the four wild-type and mutant NAs (09N1, AH N1, 09N1-s60, and AH N1+09s60. The number of pseudoparticle RNA copies produced was determined using real-time qRT-PCR with primers and a probe targeting the mRNA for the CMV–GFP reporter gene packaged in the pseudoparticles.

**Figure 3:**
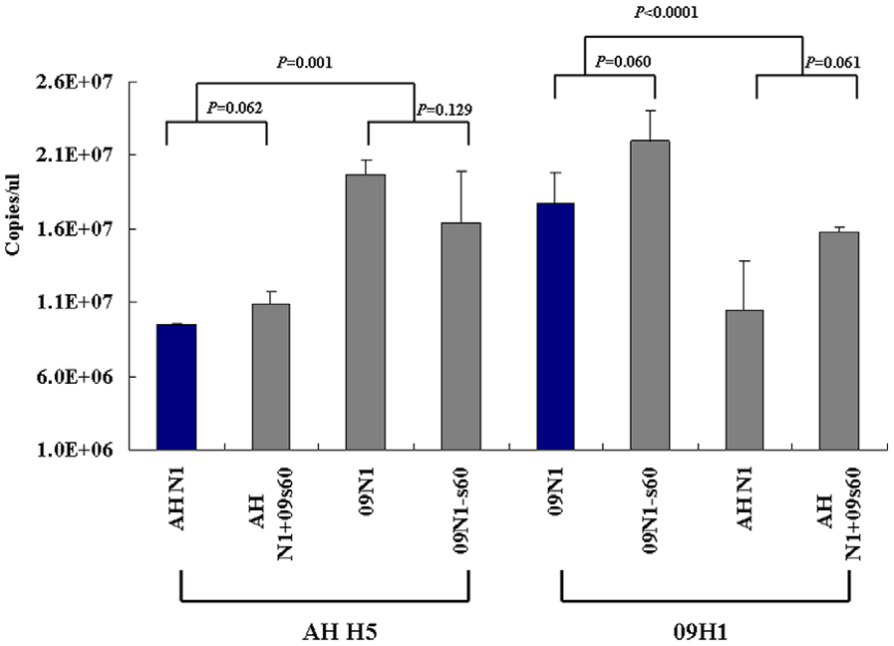
Influence of NAs on viral release. The AH N1, AH N1+09s60, 09N1, and 09N1-s60 were combined with AH H5 or 09H1 HA to generate eight pseudoparticles, and the release efficiencies of the resulting pseudoparticles were assessed by quantitative PCR (*n* = 4).

The pseudoparticle copy number did not differ significantly between AH H5::AH N1 and AH H5::AH N1+09s60 (*P* = 0.062), AH H5::09N1 and AH H5::09N1-s60 (*P* = 0.129), 09H1::09N1 and 09H1::09N1-s60 (*P* = 0.060), and 09H1::AH N1 and 09H1::AH N1+09s60 (*P* = 0.061; Fig. 3). On the other hand, for the AH H5-harboring pseudoparticles, approximately twice as many pseudoparticles were released by 09N1 and 09N1-s60 as by AH N1 and AH N1+09s60 (*P* = 0.001; Fig. 3, left); similarly, for 09H1-harboring pseudoparticles, more pseudoparticles were released by 09N1 and 09N1-s60 than by AH N1 and AH N1+09s60 (*P*<0.0001; Fig. 3, right). Taken together, these results show that 09N1 is more effective at pseudoparticle release than AH N1 and that the 20-aa deletion/insertion in 09N1 and AH N1 does not alter this relationship.

### NA activity assessment

**Figure 4:**
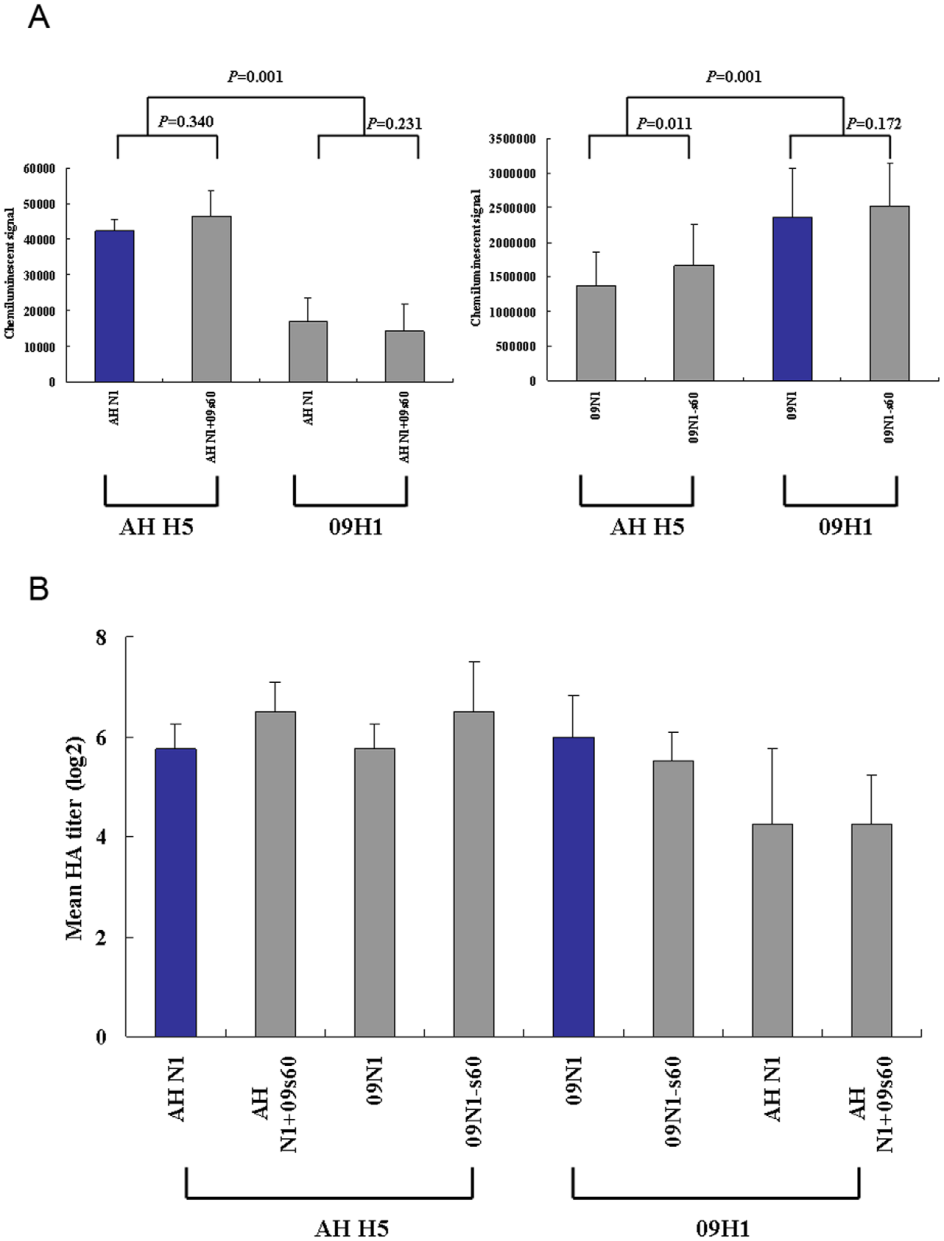
NA and hemagglutination activities of pseudoparticles. (A) NA assay. Pseudoparticles were normalized for RNA copy number and analyzed for NA activity using a chemiluminescent substrate (*n* = 4). (B) Hemagglutination assay. Pseudoparticles were serially diluted 1:2 in a 96-well plate. Hemagglutination activity is expressed as the mean HA titer (log2 HA units/50 µl) of each pseudoparticle (*n* = 4).

Despite the considerable amount of variation known to exist in the NA stalk region, little research has focused on the effects of these variations on NA activity. Therefore, we examined the NA activity of 09N1, 09N1-s60, AH N1, and AH N1+09s60 not only in their native combinations (AH H5::AH N1, AH H5::AH N1+09s60, 09H1::09N1, and 09H1::09N1-s60) but also in mismatched pseudoparticles (09H1::AH N1, 09H1::AH N1+09s60, AH H5::09N1, and AH H5::09N1-s60). As we reported previously, the NA activity of 09N1 was much higher than that of AH N1 (17, 18). When combined with AH H5, AH N1 and AH N1+09s60 exhibited NA activities of 42310±3335 and 46580±7054 chemiluminescent units, respectively, indicating that the 20-aa insertion had little effect on AH N1 activity (*P* = 0.340). The insertion also had little effect when AH N1 and AH N1+09s60 were combined with 09H1; the resulting NA activities were 16770±6573 and 14230±7636 chemiluminescent units, respectively (*P* = 0.231). AH N1 and AH N1+09s60 exhibited higher activity in native pseudoparticles (those harboring AH H5) than in mismatched pseudoparticles (those harboring 09H1; *P* = 0.001; Fig. 4A, left).

When combined with AH H5, 09N1 and 09N1-s60 exhibited NA activities of 1373620±478097 and 1657100±598128 chemiluminescent units, respectively, indicating that the 20-aa deletion significantly increased the activity of 09N1 (*P* = 0.011). However, when combined with 09H1, the deletion in 09N1 did not significantly alter its activity; in this case, 09N1 and 09N1-s60 had NA activities of 2367470±698727 and 2518920±619100 chemiluminescent units, respectively (*P* = 0.172). The NA activities of 09N1 and 09N1-s60 were higher in combination with 09H1 than with AH H5 (*P* = 0.001; Fig. 4A, right), again indicating a higher activity in the native pseudoparticles than in the mismatched pseudoparticles.

### Hemagglutination assays

To examine the influence of the wild-type and mutant NA proteins on their partner HA proteins, the pseudoparticles were normalized for RNA copy number and assayed for hemagglutination activity, as described previously [18], [19]. The mean HA titers were similar for all native, mismatched, and mutant combinations of AH H5 (AH H5::AH N1, AH H5::AH N1+09s60, AH H5:09N1, and AH H5:09N1-s60; Fig. 4B, left), indicating that neither the viral origin of the NA partner nor the NA deletion/insertion mutation affected the hemagglutination ability of AH H5. For combinations of 09H1 (09H1::AH N1, 09H1::AH N1+09s60, 09H1::09N1, and 09H1::09N1-s60), the mean HA titers were somewhat higher with 09N1 and 09N1-s60 than with AH N1 and AH N1+09s60, but neither the deletion in 09N1 nor the insertion into AH N1 caused a significant change in the hemagglutination titer (Fig. 4B, right).

### Infectivity assays

To more closely examine the role of NA, and the NA stalk in particular, we determined the infectivity of pseudoparticles containing the eight native and mismatched combinations of HA and wild-type or mutant NA. The pseudoparticles were normalized to a virus titer of 1×10^4^ copies per targeting cell before assaying.

**Figure 5:**
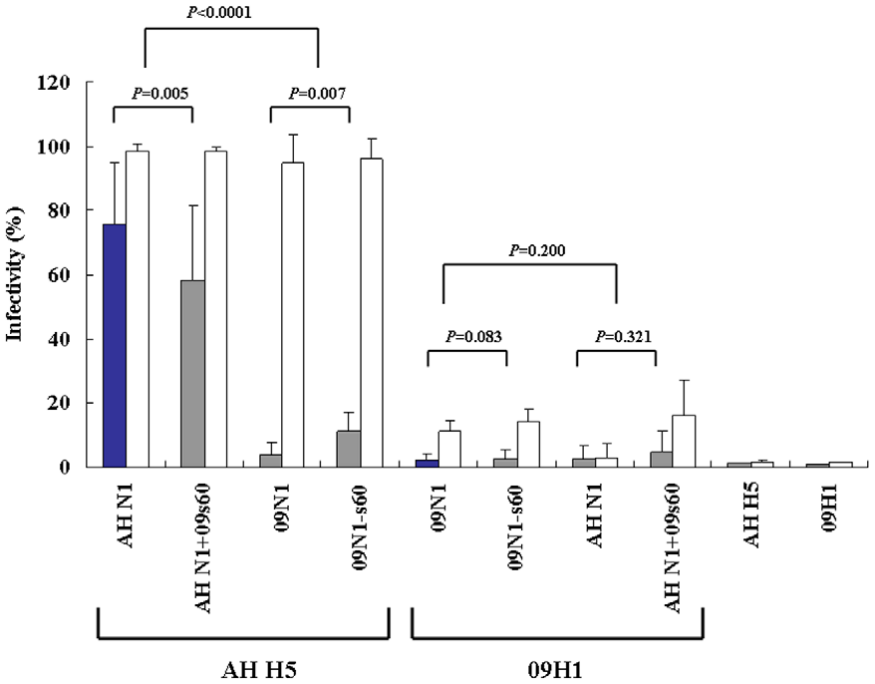
Infectivity of normalized pseudoparticles. Infectivity is presented as the mean ± SD percentage of infected cells (*n* = 4). Blue bars, infectivity of pseudoparticles comprising native combinations of HA and NA (09H1::09N1 and AH H5::AH N1); gray bars, infectivity of mismatched pseudoparticles; white bars, infectivity of pseudoparticles in the presence of oseltamivir.

The infectivity of the native wild-type combination AH H5::AH N1 was 76.0±18.6%, a value almost 40 times that of the other native wild-type combination (09H1::09N1), which had an infectivity of 1.95±1.48% (Fig. 5; blue bars). The infectivity of the native mutant combination AH H5::AH N1+09s60 (58.4±22.3%) was significantly lower than that of the native wild-type combination AH H5::AH N1 (76.0±18.6%; *P* = 0.005), suggesting that the insertion of s60 into AH N1 reduced the viral infectivity of the H5N1 pseudoparticle. On the other hand, deletion of s60 from the 09N1 stalk region did not alter its infectivity; the wild-type (09H1::09N1; 1.95±1.48%) and mutant (09H1::09N1-s60; 2.75±2.38%) native H1N1 pseudoparticles did not differ significantly in infectivity (*P* = 0.083; Fig. 5).

For the mismatched 09H1 pseudoparticles, the infectivities of the wild-type (09H1::AH N1; 2.75±1.91%) and mutant (09H1::AH N1+09s60; 4.70±3.37%) pseudoparticles were similar (*P* = 0.323; Fig. 5). In contrast, the mismatched AH H5 pseudoparticles differed in infectivity; that of the mutant (AH H5::09N1-s60; 11.05±5.07%) was significantly higher than that of the wild-type (AH H5::09N1; 4.02±3.18%) pseudoparticle (*P* = 0.007; Fig. 5). This result indicates that the deletion of s60 from 09N1 increased the viral infectivity of the mismatched AH H5::09N1 pseudoparticle. The control H5 and H1 pseudoparticles, which were generated without NA, exhibited the lowest infectivities (1.22±0.25 and 0.75±0.16%, respectively; Fig. 5).

### The robust NA activity of the 2009H1N1 virus is responsible for the high sensitivity of the virus to oseltamivir

We previously reported that oseltamivir boosts the infectivity of the 2009 pandemic H1N1 virus dramatically *in vitro* and suggested that its robust NA activity was the major cause of this effect [17], [20]. Here, we examined the effect of oseltamivir on the infectivity of pseudoparticles containing the eight native and mismatched combinations of HA and wild-type or mutant NA (Fig. 5, white bars). Oseltamivir significantly enhanced the infectivity of all of the pseudoparticles save the mismatched wild-type 09H1::AH N1, which is barely infectious. Among the native AH H5-harboring pseudoparticles, the infectivity of AH H5::AH N1 and AH H5::AH N1+09s60 was high, even in the absence of oseltamivir, but oseltamivir provided a further boost, suggesting that the NA activity of AH N1 is lower than that of 09N1, causing AH N1 to have only a slight effect on HA entry in the presence of oseltamivir. Oseltamivir boosted the infectivity of the mismatched AH H5 pseudoparticles AH H5::09N1 and AH H5::09N1-s60 by 15 and 8 times, respectively, suggesting that the higher the NA activity, the higher the infectivity-enhancing effect of oseltamivir.

### Expression of wild-type and mutant NA proteins

**Figure 6:**
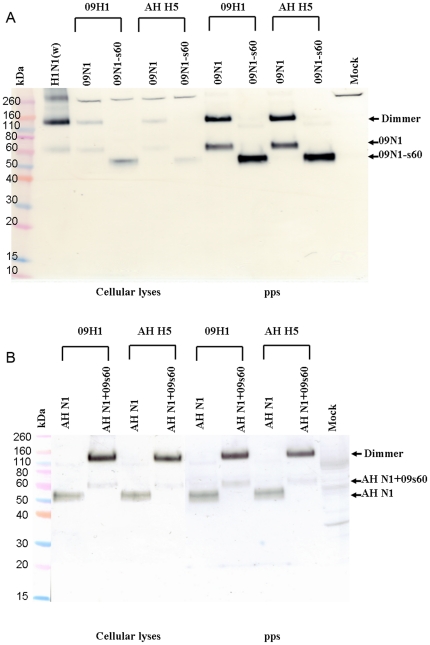
Western blot analysis of expression of NA in pseudoparticle producer 293 cells and NA incorporation into pseudoparticles. (A) Pseudoparticles and their producer 293T cell lysates were analyzed for 09N1 and 09N1-s60 expression. Wild-type H1N1 virus A/California/7/2009 (300 ng) was used as a positive control. Mock, normal 293T cell lysate; pps, pseudoparticles. (B) Pseudoparticles and their producer 293T cell lysates were analyzed for AH N1 and AH N1+09s60 expression. Mock, normal 293T cell lysate; pps, pseudoparticles.

The expression of the 09N1, 09N1-s60, AH N1, and AH N1+09s60 NA proteins in pseudoparticle producer cells was analyzed by Western blotting using a 2009 pandemic H1N1 wild-type virus isolate as a control. As shown in Figure 6A, the wild-type NA from this virus was detected primarily as a ∼120-kDa dimer but also as a tetramer and, in lesser amounts, as a ∼60-kDa monomer. The molecular mass and glycosylation pattern of 09N1 expressed in pseudoparticle-infected 293T cells appeared identical to those of the wild-type NA, but the proportion that was tetrameric was smaller. The 09N1-s60 protein appeared to be a ∼50-kDa monomer, suggesting that the ∼10-kDa deletion of s60 abolished NA oligomerization but did not alter its glycosylation pattern. The expression efficiency for the 09N1 and 09N1-s60 proteins was very similar in all pseudotype combinations, suggesting that the s60 deletion in the 09N1 stalk region did not alter its expression.

As shown in Figure 6B, the molecular masses of AH N1 and AH N1+09s60 expressed in pseudoparticle-infected 293T cells were ∼50 and ∼60 kDa, as expected. These two proteins had similar expression efficiencies in all pseudotype combinations, suggesting that the insertion in the AH N1 stalk region did not alter its expression. Notably, AH N1 was almost entirely monomeric, whereas AH N1+09s60 was primarily dimeric. Thus, the s60 insertion favored dimerization but did not alter the glycosylation pattern.

### Expression of HA proteins

**Figure 7:**
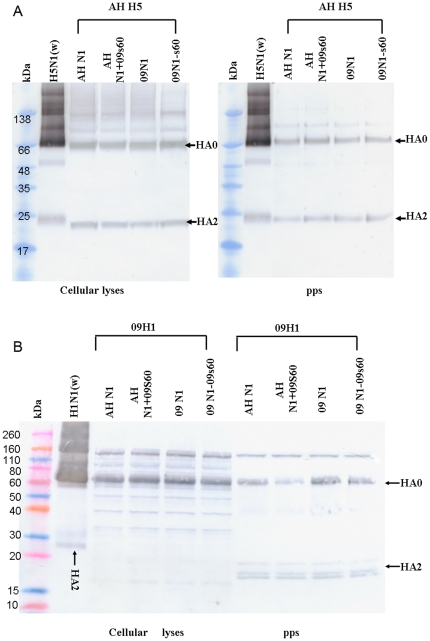
Western blot analysis of expression of HA in pseudoparticle producer 293 cells and HA incorporation into pseudoparticles. (A) Pseudoparticles and their producer 293T cell lysates were analyzed for AH H5 expression. Wild-type H5N1 virus A/Vietnam/1194/2005 (300 ng) was used as a positive control. (B) Pseudoparticles and their producer 293T cell lysates were analyzed for 09H1 expression. Wild-type H1N1 virus A/California/7/2009 (300 ng) was used as a positive control.

To confirm that the HA proteins were expressed efficiently in all pseudotype combinations, and to assess any possible effects of mismatched or mutant NAs on HA expression in pseudoparticle producer cells, we analyzed HA expression using Western blotting. Cells infected with any of the pseudotype combinations expressed AH H5 with a glycosylation pattern and a molecular mass similar to those of HA from purified, inactivated wild-type H5N1 virus (Fig. 7A, left). Furthermore, AH H5 was expressed in similar amounts in all combinations, suggesting that neither the viral origin of the NA partner (native or mismatched) nor the s60 insertion/deletion affected its expression.

Like AH H5, 09H1 was expressed with a glycosylation pattern and a molecular mass similar to those of HA from purified, inactivated wild-type pandemic H5N1 virus in all combinations, and the viral origin of the NA and the s60 insertion/deletion did not affect its expression (Fig. 7B, left).

### Evaluation of HA and NA incorporation into pseudoparticles

To evaluate the incorporation of HA and NA into the various pseudoparticles, the levels of expression of HA and NA in pseudoparticle batches of 1 million were analyzed by Western blotting.

The AH H5 protein was expressed at similar levels in all pseudotype combinations, and its molecular weight and a glycosylation pattern in all combinations were similar to those of purified wild-type HPAI H5N1 HA (Fig. 7A, right). Similarly, HA0 of 09H1 was expressed at similar levels in all pseudotype combinations and with a molecular weight and a glycosylation pattern similar to those of HA from wild-type 2009 pandemic H1N1 (Fig. 7B, right). Notably, HA2 of the pseudoparticles was a bit light than that of wild-type 2009 pandemic H1N1, this may due to difference of the glyscosylation efficiency in variant generating approaches, wild type was inoculated in egg, while, pseudoparticles were produced in 293T cells. Together with the transmission electron microscopy observation, our data suggest that the AH H5 and 09H1 HA proteins are incorporated with similar efficiency into all pseudotype combinations and that the s60 insertion/deletion does not affect this efficiency.

As shown in Figure 6A, the molecular mass of the 09N1 NA protein in pseudoparticles harboring 09H1 or AH H5 was identical to that of the NA from wild-type 2009 pandemic H1N1 virus; the monomer and dimer molecular masses were ∼60 and ∼120 kDa, respectively. The molecular mass of the 09N1-s60 NA protein in pseudoparticles harboring 09H1 or AH H5 was ∼50 kDa. The 09N1 and 09N1-s60 proteins exhibited similar incorporation efficiencies with 09H1 and AH H5, suggesting that the incorporation of 09N1 was unaffected by the s60 deletion in its stalk region. Similarly, when expressed with 09H1 and AH H5, the molecular masses of AH N1 and AH N1+09s60 were ∼50 and ∼60 kDa, as expected (Fig. 6B). These proteins exhibited similar incorporation efficiencies in AH H5- and 09H1-containing pseudoparticles, suggesting that the incorporation of AH N1 NA was unaffected by the s60 insertion.

Notably, the NA proteins lacking s60, the 20-aa stalk segment (AH N1 and 09N1-s60), produced almost no dimers, whereas the NA proteins containing s60 (09N1 and AH N1+09s60) existed primarily as dimers; thus, the 20-aa segment appears to be the critical domain for dimer formation and does not seem to affect glycosylation (Fig. 6B).

## Discussion

In this report, we focused on the NA enzymes of two influenza A virus strains that have had catastrophic effects, HPAI H5N1 and pandemic 2009H1N1. This latter virus originated in birds, jumped to swine, and then became the human pandemic strain 2009H1N1 [17]–[21], [24]. H5N1 jumped to humans directly from birds [25]. The NAs of both of these viruses display human host adaptations, but of the two, 09N1 is more robust and is thus more efficient at viral release. During the 2009H1N1 entry stage, 09N1 might be a powerful “block” against viral entry, thus making the virus sensitive to oseltamivir. Although many virologists believe that NA is tetrameric [1], [2], [9], [10], [26], we found that the 09N1 protein of the wild-type virus was primarily dimeric, although some of it was tetrameric form, and a much smaller amount was monomeric western blot analysis. Interestingly, although 09N1 in pseudoparticles was also primarily dimeric, a substantial fraction of it was monomeric, and very little was tetrameric western blot analysis.

Like the AH N1 NA protein, the 09N1-s60 NA protein, which lacks a 20-aa segment (residues 49–68; designated s60) of the 09N1 stalk region, was primarily monomeric, with a small amount of the dimeric form. When the s60 segment of 09N1 was inserted into the AH N1 stalk region, the resulting AH N1+09s60 protein was primarily dimeric, with some of the monomeric form also present. Thus, the stalk region is critical for dimer formation, which may occur *via* oxidation of Cys49, and the dimer is the major functional unit of NA. Unlike HA, NA was poorly glycosylated despite the presence of as many as five potential glycosylation sites, including the four sites in the 20-aa stalk region.

Of the 29 aas in the NA transmembrane domains, there are 8 aas difference (27.5%) which is much higher than the that of other regions, although reports indicated that the transmembrane domain of NA was critical in apical transport [27], [28], as far as we know, no report showed that the transmembrane domain was critical for pseudoparticle's budding. At the budding stage, pseudoparticles were secreted by the gag-pol protein derived from murine leukemia virus, the NA could only help pseudoparticle release by cleaving terminal sialic acids from oligosaccharide side-chain receptors that bind the HA. The more activity of NA, the more pseudoparticles released. Of the NA activity, 09 NA is almost 100 times higher than that of AH NA, the extreme high NA activity of 2009 pandemic H1N1 NA was confirmed by our previous reports [17], [18]. Together with our NA incorporation analysis, the NA activity is determined by the character of each NA, particular the ectodomain not the transmembrane domain.

Comparison of all of the native and mismatched pseudoparticles showed that those harboring AH H5 had a much higher infectivity than those harboring 09H1. This finding suggests that the HPAI H5N1 virus also has a high infectivity, and this characteristic may underlie the dire clinical outcomes of H5N1-infected patients. Of the AH H5-harboring pseudoparticles, higher infectivity was observed for AH H5::AH N1 than for AH H5::AH N1+09s60, and higher infectivity was observed for AH H5::09N1-s60 than for AH H5::09N1, suggesting that AH H5 partners more effectively with NA proteins that have a short stalk or that exist as monomers. Of the lower infectivity of all pseudoparticles harboring 09HA, the diversity in glycosylation might be a reason since we observed that HA2 of our pseudoparticles was poorly glycosylated (Figure 7B right). We did not observe any influence of the NA stalk on the infectivity of 09H1-harboring pseudoparticles, but we cannot discount the possibility that any such influence might have been masked by the lower infectivity of 09H1.

When we compared the NA activities of all eight pseudoparticle combinations of AH H5 and 09H1 with 09N1, 09N1-s60, AH N1, and AH N1+09s60, we found that the s60 insertion or deletion did not appear to affect NA activity except when AH H5 was combined with 09N1. Because of this confusion, we doubled the methodology; the NA-Star NA Inhibitor Resistance Detection Kit is extremely sensitive and hence lacks the desired reproducibility and specificity.

Oseltamivir is a NA inhibitor designed to bind to the putative NA tetramer [10]. However, our current data demonstrate that the NA of the wild-type 2009 pandemic H1N1 is primarily dimeric and that it exists in both dimeric and monomeric forms in pseudoparticles. This finding, in conjunction with our previous finding that NA dramatically inhibits NA activity [17], casts doubt upon the accepted pharmacologic mechanism of action of oseltamivir. We previously noted that oseltamivir boosts the infectivity of influenza virus [17]; the present comparison of NA activities in pseudoparticles pairing AH H5 and 09H1 partnered with native and mismatched NA proteins shows clearly that the infectivity-enhancing effect of oseltamivir correlates positively with the NA activity. Therefore, we feel we must again raise an alarm concerning the use of oseltamivir to treat influenza A in clinical practice: when the patient's lung has a high viral load, oseltamivir administration might enhance the infectivity of the virus, particularly if the virus has a high NA activity to begin with. The resulting robust pulmonary immune response could result in unexpected lung failure.

Retrovirus-based influenza HA/NA pseudoparticle systems have been demonstrated to accurately represent the biology of the corresponding wild-type viruses [17]–[21], [29], [30]. For research on HPAI viruses, the use of pseudoparticle systems eliminates not only routine biosafety issues but also the possibility of production of a manmade, highly pathogenic virus. Pseudoparticles containing only HA and NA provide a convenient model for research on HA/NA matching patterns, viral release, viral entry, neutralizing antibodies, and many other biological features involving HA and NA. Although this system cannot completely represent the entire virus generated by reverse genetics, it has provided us with a safe and convenient platform for our HA/NA studies.

Due to the lacking of antibody against AH NA, we added a 6 x His-encoding sequence to the C-terminal ends of the AH NA and AH N1+09s60 coding sequence to facilitate Western blot analysis. We have assessed any possible influence of His tag on NA's function, there was no difference in NA activity and cooperation with HA between the pseudoparticles harboring NAs with or without His tag (data not showed).

The HAs of HPAI viruses have multiple basic amino acids at their cleavage sites (RERRRKKR) and are cleaved by ubiquitous proteases in a wide range of organs, resulting in lethal systemic infection [1]. We observed that pseudoparticles harboring AH H5 were more infective than those harboring 09H1, but whether the presence of the HPAI HA cleavage site enhances the infectivity of HPAI H5N1 viruses is unknown. We are currently performing experiments to address this issue.
